# Cost-effectiveness analysis of active day patient treatment, an interdisciplinary pain self-management program

**DOI:** 10.1097/PR9.0000000000001306

**Published:** 2025-09-10

**Authors:** Anonnya Rizwana Chowdhury, Deborah Schofield, Rupendra Shrestha, Michael Nicholas

**Affiliations:** a Pain Management Research Institute, Sydney Medical School, University of Sydney, Sydney, Australia; b Centre for Economic Impacts of Genomic Medicine (GenIMPACT), Macquarie Business School, Macquarie University, Sydney, Australia

**Keywords:** Chronic pain, Interdisciplinary, Pain self-management, Cost-effective, Labour force participation, Quality of life

## Abstract

**Introduction:**

Active day patient treatment (ADAPT) is an interdisciplinary, multimodal pain management program for patients with disabling chronic pain at the Pain Management & Research Centre (PMRC), Royal North Shore Hospital in Sydney, Australia.

**Objective:**

The aim of this study was to analyse the cost-effectiveness of ADAPT using hospital administrative and patient reported >12-month follow-up data. Patients' preprogram labour force participation, health care utilization costs, and quality of life outcomes were compared with outcomes >12 months after participating the program.

**Methods:**

This retrospective cohort study included 61 patients who completed ADAPT (including follow-up) between 2014 and 2017 at the PMRC. Primary outcome measures were labour force participation in patients' average weekly earnings (n = 61) and incremental cost-effectiveness ratio (ICERs) (n = 53) based on the health utility score derived from assessment of quality of life (AQoL-8D). Pain-related health care utilization and costs before and >12 months after the program were assessed to calculate the ICER.

**Results:**

We estimated patients were earning $628.99 per week at >12 month after participating in ADAPT comparing to $539.54 at baseline. The ICER was $20,228.76 (95% CI: $14,176.25-$27,826.33) per quality adjusted life years gained.

**Conclusion:**

Results from this study indicated that, patients with chronic pain >12 months after participating in ADAPT reported higher quality of life scores along with reduced health care utilization costs. However, more robust and exhaustive studies, especially prospective studies, are required to confirm these findings.

## Abstract 2

Supplemental Digital Content is Available in the Text.

Results from this study indicated higher quality of life and reduced health care utilization costs in patients with chronic pain >12 months after participating in active day patient treatment.

## 1. Introduction

Many of those with chronic pain conditions report impaired quality of life, affecting both physical and mental wellbeing. Patients attending health services with chronic pain often have difficulties performing daily activities, including paid work and home duties.^23^ Chronic pain has also been associated with higher societal costs. These include lost productivity in the working age population, as well as health care utilization costs, with hospital visits due to chronic pain–related issues increasing over recent decades.^14,17,24^

Intensive, interdisciplinary pain management programs for people with disabling chronic pain have been recommended since the 1970s over pharmacological and surgical interventions,^11,12,14^ and there is some evidence they can be cost-effective for the management of chronic pain in adults.^3,5,21,22^ However, the National Institute of Health and Care Excellence (NICE) guideline of Chronic Pain (2021) has suggested that demonstrating effectiveness or cost-effectiveness of pain management program can be challenging when treating chronic primary pain.^30^ Studies performed in the United Kingdom, United States, and Australia have indicated reduction in patients' opioid dependence and enhanced levels of function after participating in the outpatient interdisciplinary chronic pain self-management programs.^6,18,33,39,43^ But, understandably, funders of these services should expect to see evidence of their cost-effectiveness. Some studies have demonstrated the cost-effectiveness of similar approaches to pain self-management delivered online, but the cohorts treated in these studies represent a much more functional population than those typically seen in tertiary referral centres.^7,8,16,34^ These differences make it difficult to generalise their findings to the management of people who are significantly disabled, often quite depressed and medication dependent. For example, unlike patients referred to tertiary pain centres by their treating doctors, the participants in the online programs were recruited by advertising, most were working and relative few reported being unable to work due to their pain. Also, medication use, particularly use of opioids, was not a common feature of these cohorts. So, we still lack evidence that intensive programs for the more disabled patients are cost-effective.

This paper concerns a 3-week (15 day) interdisciplinary pain management program called active day patient treatment (ADAPT) that has been conducted at the Royal North Shore Hospital in Sydney, Australia, since 1994. Patients referred to ADAPT are patients with chronic pain who have already exhausted all other forms of intervention recommended by their doctors who have referred them to a multidisciplinary pain centre often as a last resort. Previous studies of outcomes from this program have indicated that it is effective in substantially reducing the use of opioid medication by participants over a 1-year follow-up, as well as in achieving significant improvements in daily function, mood and cognitive variables.^30,31,33^ However, there is only short-term data on the program's cost-effectiveness.^4^

The aim of this paper was to assess the cost-effectiveness of the ADAPT program from a health care perspective based on ADAPT patients' health care services utilization cost and quality of life outcomes over an extended period. This study also analyses the changes in labour force participation (LFP) of patients at >12 months after participating in the ADAPT program in average weekly earnings.

## 2. Methods

### 2.1. Participants and intervention

This is a retrospective cohort study of 61 patients who completed the ADAPT program at the Pain Management & Research Centre (PMRC) in the Royal North Shore Hospital (RNSH) in Sydney, Australia and were followed up >12 months later.^4^ These patients are a subgroup (26.5%) of 230 ADAPT patients who previously completed ADAPT between 2014 and 2017.^4^ A detailed description of the ADAPT program, a multimodal program, has been published elsewhere.^4,31^

Ethical approval was obtained from the Northern Sydney Local Health District HREC (Human Resource Ethics Committee), and consent was obtained from all participating patients.

### 2.2. Data

Assessment measures for the LFP and health care utilization were completed by patients before the program. Patients' pre-ADAPT labour force participation and health care utilization cost data were extracted from the electronic Persistent Pain Outcomes Collaboration (ePPOC) database.^32^ Electronic Persistent Pain Outcomes Collaboration is an Australian initiative that has become a valuable resource for pain researchers and clinicians since its commencement in 2013. Active day patient treatment patients' clinical and health care resource use data are administered electronically as part of the ePPOC initiative.

**Figure 1.**
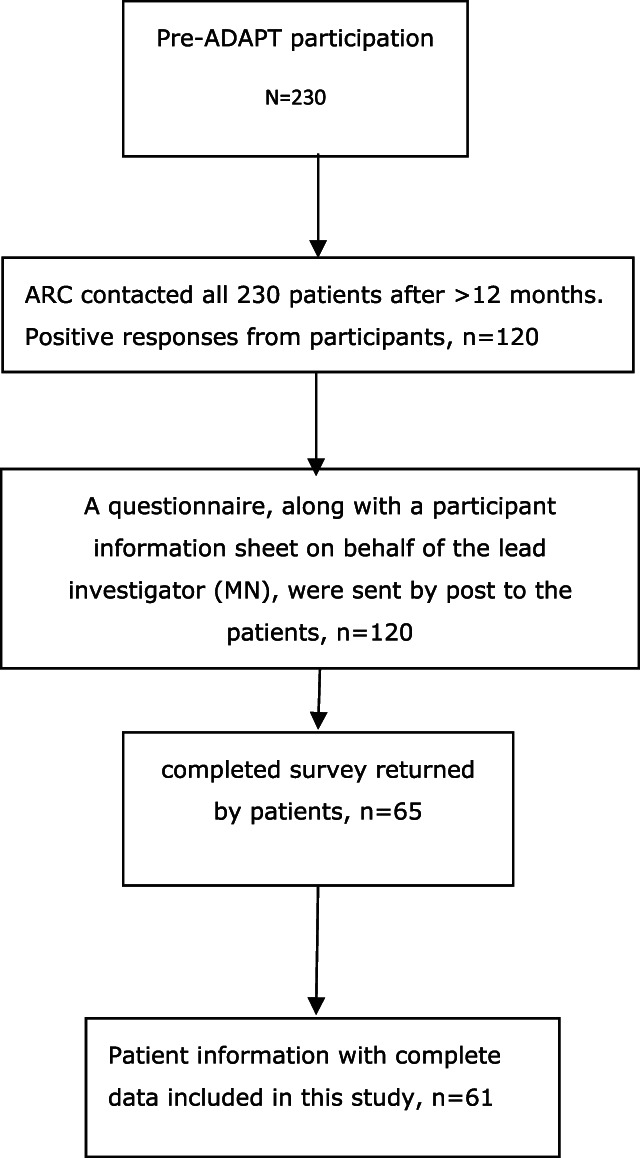
Availability of data at pretreatment and >12-month follow-up.

We conducted a >12-month follow-up survey to collect patients' LFP, quality of life (both pre and post program), and health care utilization cost data. We compared patients' pretreatment and >12-month follow-up data for this analysis. We were able to contact 120 patients of the above 230 patients through telephone for their participation in the >12-month follow-up survey. A questionnaire along with a participant information sheet on behalf of the lead investigator (M.N.) were sent by post to the patients who agreed to participate. Out of 120, 65 (54.2%) patients returned their completed survey. Patient ID was missing in 2 responses; therefore, we could not identify 2 patients for those responses. The survey questions captured ADAPT patients' LFP and health care utilization >12 months after participating the program, and quality of life using the AQoL-8D (Assessment of Quality of Life). Active day patient treatment patients' quality of life was not assessed at pre-ADAPT. Therefore, we asked the patients to provide their quality of life data retrospectively. Labour force participation data were available for 61 patients, and cost data were available for 60 patients (see Fig. 1 for data availability at pretreatment and >12-month follow-up).

#### 2.2.1. Health care utilization and cost

We collected pre-ADAPT data related to the cost of the ADAPT program and patients' health care services utilization over 3 months (including number of visits to a general practitioner, pain specialists, allied health professionals such as physiotherapists, hospital emergency department visits, hospital admission, and diagnostic tests) from the RNSH patient administrative data. We collected patients' health services utilization data over the last 12 months at our >12-month follow-up survey. We converted the health care utilization (HCU) data over 12 months to HCU over 3 months to be able to compare the cost before ADAPT and at >12-month follow-up. Patients were funded by either Medicare or workers' compensation (or motor-accident) insurance. We collected the costs of Medicare items and workers' compensation items from publicly available Medicare data and data available from the Independent Hospital Pricing Authority (IHPA) and State Insurance Regulatory Authority, New South Wales (SIRA NSW) Web sites. We calculated the costs as per the 2019 schedule.^9,19,29^

#### 2.2.2. Labour force participation

We used patient's preprogram work status data recorded as “full time employment,” “part time employment,” “retired,” “unemployed due to pain,” “unemployed (not due to pain),” “home duties,” “on leave from work,” “student,” “voluntary work,” “retraining” or “limited hours and/or duties” and the number of hours worked for those in “part time employment,” work/study hours affected by pain, work type affected by pain based on hospital administrative data from the ePPOC database. For our analysis, we used paid employment data on “full time employment,” “part time employment,” and “limited hours and/or duties” at preprogram. We collected data on hours worked at >12-month follow-up.

We conducted an LFP analysis based on patients' participation in paid employment. We asked the patients: “If you are currently in paid work, about how many hours each week do you work?” The Australian Bureau of Statistics reported that full-time workers were working 38 hours per week and their average weekly income was $1,634.7 in May 2019.^1^ We assumed patients who were working, on average 15 hours per week, were working part time. We further assumed patients were working 8 hours per week for “limited hours of work.” Thus, we included complete data on full time, part time, and limited hours to calculate labour force participation in weekly income and productivity gain (hours worked per week per patient) > 12 months after completing ADAPT.

#### 2.2.3. Quality of life measure

Quality-adjusted life years (QALYs) are commonly used in the economic evaluation studies as a unit of cost-utility measure for the analysis of health interventions affecting psycho-social health.^37^ Quality-adjusted life years can estimate patients' quality of life. Quality-adjusted life years are defined as life years times the health state utility measured on a 0 (death) to 1 (full health) scale.^27^ Utilities have been increasingly measured by researchers and decision makers using a multi-attribute utility (MAU) “instrument” such as AQoL-8D (Assessment of Quality of Life).^2,38^ The AQoL-8D questionnaire consists of 35 items in 8 dimensions. These dimensions include (1) 3 physical dimensions (independent living, pain, and senses) and (2) 5 psycho-social dimensions (mental health, happiness, coping, relationships, and self-worth).^34,36^ The AQoL-8D is a set of questions and response categories (the descriptive system or classification) and a corresponding set of utility weights.^2^

AQoL-8D was not included as a QoL measure at baseline to be completed by the patients participating the ADAPT program. Therefore, we retrospectively collected data for baseline by survey, along with the quality of life data at >12 months.

#### 2.2.4. Cost-utility analysis

An incremental cost-effectiveness ratio (ICER) was calculated based on AQoL-8D and HCU costs. The ICER was calculated as additional cost per unit improvement in the AQoL-8D utility score after ADAPT for the QoL:

ICER(ADAPT)=Increase in Cost/Improvement in QoL

where, Increase in Cost = (ADAPT intervention cost + total HCU cost at >12-month follow-up) − (total HCU cost before ADAPT).

From the ICER, we estimated the additional costs per QALY gained by ADAPT patients. 95% confidence intervals (CI) for ICER analysis were estimated using bootstrapping techniques by generating 1000 replicated datasets.

**Figure 2.**
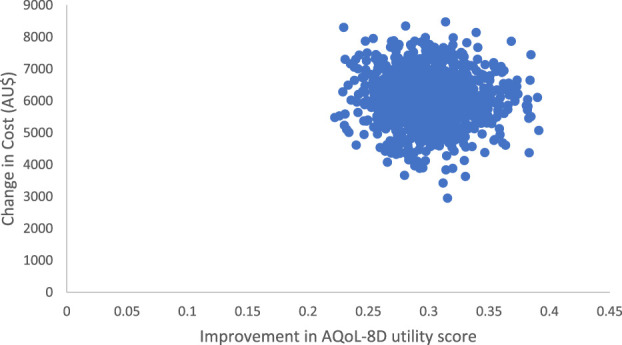
Cost-effectiveness plane based on AQoL-8D utility score; before and 1 month after participating ADAPT-health care perspective. ADAPT, active day patient treatment; AQoL, assessment of quality of life.

We used bootstrapped cost utility pairs to estimate cost-effectiveness planes (also known as, Cost-utility Planes) and cost-effectiveness acceptability curves (CEAC or also known as, cost-utility acceptability curves).^16,21,22,34,42^ To assess the variability of this estimate, we plotted the changes in costs and changes in AQoL-8D mean utility scores on the CE planes (Fig. 2) for each of the 1000 bootstrap estimates.

### 2.3. Statistical Analysis

We used a paired sample *t* test to compare changes in number of hours worked per week and HCU costs. AQoL-8D utility scores for pre-ADAPT and >12-month follow-up were calculated using IBM SPSS Statistics.^28^ Data processing for LFP and HCU were conducted in Microsoft Excel. Bootstrapped replicates of costs per QALY gained estimated after >12-month follow-up were conducted in SAS Studio version 9.4.

## 3. Results

### 3.1. Baseline characteristics

Of the 61 (26.5% of the 230 patients from our previous study) patients' data collected at the >12-month survey, 6 patients did not have complete data for AQoL-8D utility score. One patient did not have health care utilization data, leaving a total sample at 53 for the ICER analysis. We compared patients' pretreatment and >12-month follow-up data for this analysis. Participants were adults aged 16 to 65 years (57% female and 43% male) with pain lasting more than 3 months. We included 61 patients' complete LFP data in this analysis.

Table 1 shows characteristics of the participants.

**Table 1:** Characteristics of the participants.

Age	n = 227*	n = 61
	n (%)	n (%)
Age group (y)		
16–20	5 (2)	4 (7)
21–35	45 (20)	7 (11)
36–50	93 (40)	28 (46)
51–65	75 (32.5)	22 (36)
66–80	11 (5)	0
81–91	1 (0.5)	0
Mean (SD)	46 (13) (min 16, max 91)	45 (12) (min = 16, max = 63)
Sex		
Female	126 (56)	35 (57)
Male	101 (44)	26 (43)
Employment		
Full time (FT)	42 (18.5)	14 (23)
Home duty (HD)	10 (4.4)	1 (2)
Limited hours (LH)	24 (10.6)	2 (3)
Leave due to pain (LP)	21 (9.3)	5 (8)
Part time (PT)	23 (10.1)	10 (16)
Retired (Ret)	20 (8.8)	3 (5)
Retraining (Retrain)	5 (2.2)	0
Student (ST)	4 (1.7)	4 (7)
Unemployed due to pain (UP)	82 (36.1)	22 (36)
Volunteer (Vol)	1 (0.4)	0
Pain and self-efficacy score		
BPI Severity (scale 0–10)		
Mean (SD)	5.99 (1.5) (min 2.25, max = 10)	5.75 (1.43) (min 2.5, max = 10)
BPI Interference (scale 0–10)		
Mean (SD)	7.12 (1.8) (min 1.28, max = 10)	6.79 (2.14) (min 1.29, max = 10)
PSEQ (scale 0–60)		
Mean (SD)	19.81 (11.6) (min 0, max 60)	21.87 (12.86) (min 0, max 54)
Utility score AQoL-8D (0–1 scale)	n = 55†	
Mean (SD)	0.25 (0.11)	

* Full sample of 227 patients who previously completed ADAPT

† Retrospectively collected at >12-mo follow-up survey.

ADAPT, active day patient treatment; AQoL, assessment of quality of life; BPI, Brief Pain Inventory; PSEQ, Pain Self Efficacy Questionnaire.

Based on the self-report scores on BPI (Brief Pain Inventory - pain severity and pain interference), PSEQ (Pain Self Efficacy Questionnaire), and AQoL-8D, the patients recruited for this study were experiencing moderately severe pain (mean = 5.75, SD: 1.43) and moderately high levels of interference (mean = 6.79; SD: 2.14) in daily activities due to pain, low pain self-efficacy (mean = 21.87; SD: 12.86), and low quality of life (mean utility score = 0.25; SD: 0.11). These scores would place the sample at close to the median of patients generally seen in tertiary level pain services in Australia and New Zealand.^32^

Of these 61 patients, 22 (36%) were unemployed due to pain and 5 (8%) were on extended sick leave due to pain at baseline.

In comparison to the full sample of 227 patients who previously completed ADAPT, and had health outcome data, including BPI Severity and Interference, and PSEQ scores, it can be seen that the 61 recruited at long-term follow-up were reasonably representative of the full sample.^4^ AQoL-8D data were not collected for this patient cohort as baseline.

### 3.2. Assessment of quality of life outcomes

Of the 60 patients who provided complete health care utilization data, 55 patients fully completed pre-ADAPT AQoL-8D questionnaire. We have the complete pre and >12-month AQoL-8D data available for 53 patients, which we used to calculate the ICER.

There were improvements in all 8 dimensions of AQoL-8D between baseline and >12 months after participating ADAPT, with the biggest improvements in happiness 0.3 points (Table 2). Other dimensions, such as independent living scores improved by 0.18, mental health value by 0.17, coping and self-worth value by 0.27, relationships value by 0.13, pain value by 0.24, and senses value by 0.10. Overall, there was a 0.20 improvement in the physical dimension and 0.19 improvement in the mental health dimension. AQoL-8D utility score improved by 0.3 (n = 53), which was statistically significant (*P* < 0.001). This is also well-above the suggested minimal clinically important difference with the AQoL of 0.06 utility points.^15^

**Table 2:** Assessment of quality of life-8D utility score before and >12 months after participating in active day patient treatment (n = 53).

Dimensions	Mean (SE) score (before ADAPT)	Mean (SE) score (12 mo after ADAPT)	Mean (SE) score improvement at 12 mo	*P*
AQoL8D Dim1 independent living value	0.56 (0.019)	0.74 (0.024)	0.18 (0.004)	<0.001
AQoL8D Dim2 happiness value	0.41 (0.022)	0.70 (0.026)	0.30 (0.004)	<0.001
AQoL8D Dim3 mental health value	0.35 (0.014)	0.52 (0.021)	0.17 (0.003)	<0.001
AQoL8D Dim4 coping value	0.42 (0.015)	0.68 (0.022)	0.27 (0.003)	<0.001
AQoL8D Dim5 relationships value	0.48 (0.005)	0.61 (0.020)	0.13 (0.003)	<0.001
AQoL8D Dim6 self-worth value	0.44 (0.023)	0.71 (0.027)	0.27 (0.004)	<0.001
AQoL8D Dim7 pain value	0.27 (0.012)	0.50 (0.029)	0.24 (0.003)	<0.001
AQoL8D Dim8 senses value	0.71 (0.029)	0.79 (0.019)	0.10 (0.003)	<0.001
AQoL8D vSuperDimPhysical value*	0.28 (0.012)	0.48 (0.025)	0.20 (0.003)	<0.001
AQoL8D vSuperDimMental value†	0.08 (0.008)	0.28 (0.024)	0.19 (0.003)	<0.001
Utility score for AQoL-8D	0.25 (0.015)	0.55 (0.030)	0.3 (0.004)	<0.001

* Super dimension physical value includes independent living, pain, and senses values.

† Super dimension mental value includes mental health, happiness, coping, relationships, and self-worth values.

ADAPT, active day patient treatment; AQoL, assessment of quality of life.

### 3.3. Labour force participation

Labour force participation data were available for 61 patients (57% female and 43% male). Patients who were working part time were, on average, working 19 hours per week at >12-month follow-up. On average, patients were estimated to earn $628.99 per week at >12 months after participating in ADAPT comparing to $539.54 at baseline (*P* = 0.277). A separate calculation was conducted using average weekly income for males and for females separately. On average, women (n = 35) were estimated to earn $528.31 and men (n = 26) were estimated to earn $773.95 per week >12 months after participating in ADAPT compared to estimated women earning $368.61 and men earning $745.41 per week at baseline. However, these changes were not statistically significant (*P* = 0.105 for women and *P* = 0.3767 for men).

### 3.4. Cost-utility analysis

#### 3.4.1. Costs

Health care utilization data were available for 60 patients (43% male and 57% female). Of these 60 patients, 40 (67%) patients were covered by Medicare and 20 (33%) patients were covered by NSW workers compensation insurance. Unit costs for health care utilization are shown in the supplementary table, http://links.lww.com/PR9/A327. The average HCU cost was significantly lower >12 months after ADAPT than before it, resulting in an average cost saving of $2,799.89 per patient over 3 months (Table 3). The reduced costs >12 months after participating in the program comprised savings from reduced General Practitioner (GP) visits ($101.93), specialist costs ($152.24), allied health costs ($173.33), emergency department (ED) costs ($280.60), hospital costs ($1,656.95), and diagnostic costs ($434.84). The cost of ADAPT at that time was $9,150.00 per patient (2019, Administrative data). Therefore, the additional cost per patient >12 months after participating in ADAPT was $6,350 ($9,150.00–2,799.89).

**Table 3:** Health care utilisation cost at before and >12 months after active day patient treatment (n = 60).

Cost (2019)	Before ADAPT Mean (SE)	>12 mo follow-up mean (SE)	*P*	Additional cost Mean (SE)
Intervention cost of ADAPT*				$9,150.00
Health care utilization cost				
GP visit	$ 227.36 ($27.82)	$ 125.43 ($29.45)	0.002	−$ 101.93 ($31.95)
Specialist visit	$ 252.73 ($53.71)	$ 100.49 ($23.78)	0.010	−$ 152.24 ($57.54)
Allied health visit	$ 252.13 ($42.52)	$ 78.80 ($22.78)	0.001	−$ 173.33 ($48.85)
Emergency department visit	$ 439.20 ($271.34)	$ 158.60 ($75.40)	0.285	−$ 280.60 ($259.99)
Hospital admission	$ 2,414.12 ($805.72)	$ 757.17 ($333.10)	0.008	−$ 1,656.95 ($616.05)
Diagnostic test	$ 678.81 ($159.91)	$ 243.97 ($51.10)	0.006	−$ 434.84 ($154.69)
Total health care utilization cost	$ 4,264.35	$ 1,464.46	0.0003	−$ 2,799.89
Additional cost per patient = intervention cost of ADAPT + (additional health care utilization cost)				$ 6,350.11

* Based on hospital administrative and 12-month follow-up data.

ADAPT, active day patient treatment.

#### 3.4.2. Cost-utility analysis of active day patient treatment

We combined 53 patients' AQoL-8D utility scores and health care utilization cost data to calculate the ICER. There was an estimated additional costs of $20,228.76 (95% CI: $14,176.25-$27,826.33) per QALYs.

An ICER of $20,228.76 would typically be considered acceptable for public funding from a health care perspective.^13,26,35^ Previous studies indicated that in Australia and the United States, the ICER threshold can be up to $50,000, and in the United Kingdom, this threshold range between £ 20,000 and £30,000.^28,41^

#### 3.4.3. Cost-effectiveness plane, cost-effectiveness acceptability curve, and uncertainty

Figure 2 shows the cost-effectiveness (CE) plane with X-axis showing improvement in AQoL-8D mean utility score at >12 months after ADAPT and Y-axis showing increased cost at >12 months after ADAPT (including the cost of the ADAPT intervention). Each point in the plots represents an estimate based on a replicated dataset. This demonstrates that while there is an additional cost to provide the ADAPT intervention, patients reported high quality of life scores.

**Figure 3.**
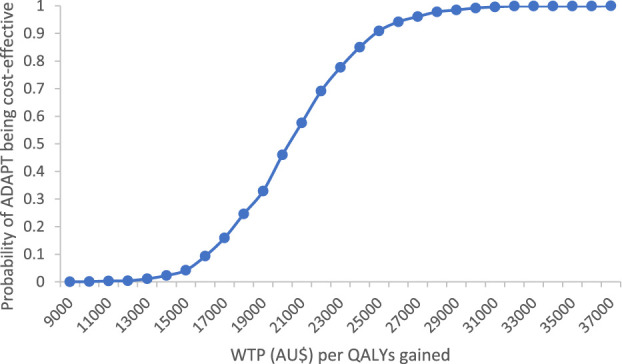
Cost-effectiveness acceptability curve based on AQoL-8D utility scores. AQoL, assessment of quality of life.

Cost-effectiveness acceptability curves show the probability that an intervention is cost-effective if the decision makers are willing to pay (WTP) to gain 1 extra unit of effect. An intervention is cost-effective if the decision makers' WTP is greater than the calculated ICER. Figure 3 indicates that there was a 85% chance that the ADAPT could be cost-effective if a WTP threshold was $24,000 for an additional quality-adjusted life year gain.

To assess applicability or generalisability of the findings of this study, a sensitivity analysis was conducted using data of the worst quartile patients (n = 17) with baseline utility score based on AQoL-8D of ≤0.18. For this group, we obtained an ICER of $21,899 (95% CI: $13,438.96–$32,961.20) per QALYs gained. While conducting the same analysis for the best quartile patients (n = 15) with utility score of ≥0.31, we obtained an ICER of $30,358.28 (95% CI: dominant to $484,975) per QALYs gained, with dominant indicating that the intervention resulted in both a net cost savings and a net increase in QALYs.^10,44^ We did not report negative ICER in 95% CI for the best quartile patients because these ratios are difficult to interpret. While conducting an ICER analysis in health economic evaluation, it is hard to identify whether an ICER is negative because the numerator is negative (the intervention is less costly than standard care) or because the denominator is negative (the intervention is less effective than standard care). Instead, in these cases, previous studies reported the intervention as dominant, meaning that the intervention results in both a net cost savings and a net increase in QALYs.^10,25,40,44^

## 4. Discussion

Chronic pain is often associated with high societal costs, mostly through increased health care utilization costs, reduced quality of life, and productivity losses.^3,23^ To date, attempts to demonstrate if these costs can be reduced by interdisciplinary pain management programs have been rare, and expert groups have recognised the challenges presented by such undertakings.^32^

The few previous economic evaluation studies on such programs have indicated that they can be effective for return to work, improved functional status, and quality of life.^3,7,10,18,20,29,39^ More recent studies of internet delivered programs have demonstrated impressive outcomes.^8,10,17,35^ However, while these have all been methodologically sound, the generalisability of their findings to more disabled cohorts of people with chronic pain is questionable. It should be noted the patients attending intensive programs like ADAPT were all referred by their treating doctors to tertiary referral pain centres for expert care. In contrast, the online programs appear to have recruited their participants by advertising online and in mass media, and the participants appeared much less disabled than those attending tertiary referral pain centres.^7,8,16,34^ The findings of the present study are broadly consistent with these studies, but we also showed that significantly reduced health care costs are achievable in a more disabled population. This cost-utility analysis indicates that a long-standing interdisciplinary pain management program (ADAPT) was more effective compared to the standard care provided before ADAPT, but naturally, these improvements came at the cost of the intervention. Even so, health care utilization costs were considerably reduced for a moderately disabled cohort of patients at >12 months after participating in ADAPT.

Previous studies indicate that chronic pain is often associated with reduced productivity, especially among workers aged 45 to 64 years.^36^ Our analysis indicates that in the 61 patients providing follow-up data, their average weekly earnings was $628.99 per week at >12 months after participating in ADAPT comparing to $539.54 at baseline. Quality of life, as measured by the AQoL-8D, the mean utility score for 53 (of the 61) patients improved by 0.29 at >12-month follow-up compared to their pre-ADAPT scores. As a quality of life measure, the AQoL-8D reflects the psychosocial dimensions of health and, therefore, can be considered as a suitable measure for evaluating health programs.^35^ Previous studies have suggested that pain is often worse for patients who are unemployed.^30^ In this analysis, on the quality of life scale (AQoL-8D), pain levels improved by 0.24 score (on a 0–1 scale) in the patients' quality of life >12 months after participating in the program.

Whether the present findings can be generalised beyond this study is always an important consideration. In part, this will depend on how representative the patient cohort is, relative to other chronic pain cohorts. As noted in the Results, the cohort studied in this paper had severity scores for pain, pain interference, and pain self-efficacy close to the median range reported in a large normative study of patients attending tertiary pain clinics in Australia and New Zealand.^36^ These scores are comparable to similar cohorts reported in tertiary-referral pain services elsewhere.^6^ Generalisability will also be influenced by the replicability of the treatment program. In this case, the outcomes on self-report measures reported here are consistent with those previously been reported from the same program with different patient cohorts since 2012,^34,35^ as well as the program in the United Kingdom on which ADAPT was based.^6^ These findings would suggest that others treating similarly disabled patients with programs consistent with ADAPT by experienced multidisciplinary staff should expect to achieve similar results.

### 4.1. Strengths and limitations

Our study has a number of strengths. We conducted a survey to collect patients' LFP, quality of life, and health care utilization cost data >12 months after participating in ADAPT. The time frame was extended from 12 to 62 months post-ADAPT, 59% of the patients responded 12 to 18 months after participating the program capturing the longer term outcome since participating in ADAPT. A study of 90 country-level health surveys reported that the recall periods ranged from 2 weeks to 14 months.^20^ Further, we used pre-ADAPT data from ePPOC, which is a standardised clinical database. We analysed long-term LFP outcomes in patients' average weekly earnings and AQoL-8D utility scores, which are increased by overall improvement in patients' life and less dependence on family members.^3^

Our study also has some limitations. First, we retrospectively collected AQoL-8D and HCU data; therefore, we cannot rule out the possibility of recall bias. Interestingly, previous studies have indicated quite reasonable concordance between recalled self-reported health utilization and registration-based records, at least for some events. In addition, we were able to collect the follow-up data for a small proportion of potential cases (26.5% of the 230 patients who previously completed ADAPT and 54.2% of the 120 patients who agreed to participate in the follow-up survey). Because of this, there is a possibility of an attrition bias in the findings, but the follow-up rate of 54% of participants who provided consents is consistent with previous studies with similar patients in this area (Huffman et al., 2017; Nicholas 2014; Williams 1999).^6,18,31^ In addition, the lack of a control group for this study does limit the possible conclusions that can be drawn.

Finally, as mentioned in the Introduction, this study took a health care service utilization perspective, rather than an individual patient perspective. Accordingly, we did not collect data on out-of-pocket expenses (like medication costs), and we did not have data on out-of-pocket medication expenses. Despite these limitations, this study adds to the limited body of evidence related to cost-effectiveness of interdisciplinary pain management programs, in quality of life, HCU costs, and LFP.

### 4.2. Conclusion

Results from this study indicated that patients with chronic pain >12 months after participating in ADAPT reported higher quality of life scores along with reduced HCU costs. However, more robust and exhaustive studies, especially prospective studies, are required to confirm these findings.

## Disclosures

M.N. receives royalties from the sale of the book: “Manage Your Pain” (ABC Books). The remaining authors have no conflicts of interests to declare.

## Supplemental digital content

Supplemental digital content associated with this article can be found online at http://links.lww.com/PR9/A327.

## References

[1] (ABS) ABoS. Average weekly earnings, Australia (Reference period May 2019). Vol. 2020. Australia, 2019. https://www.abs.gov.au/statistics/labour/earnings-and-work-hours/average-weekly-earnings-australia/may-2019.

[2] Chen G Khan MA Iezzi A Ratcliffe J Richardson J. Mapping between 6 multiattribute utility instruments. Med Decis Making 2016;36:160–75. 25840901 10.1177/0272989X15578127

[3] Chowdhury AR Graham PL Schofield D Cunich M Nicholas M. Cost-effectiveness of multidisciplinary interventions for chronic low back pain: a narrative review. Clin J Pain 2021;38:197–207. 34812772 10.1097/AJP.0000000000001009 PMC8823904

[4] Chowdhury AR Schofield D Shrestha R Nicholas M. Economic analysis of patient-related effects of an interdisciplinary pain self-management program. PAIN 2023;164:2491–500. 37326690 10.1097/j.pain.0000000000002959 PMC10578420

[5] Chowdhury AR Graham PL Schofield D Costa DSJ Nicholas M. Productivity outcomes from chronic pain management interventions in the working age population; a systematic review. PAIN 2024;165:1233–46. 38323645 10.1097/j.pain.0000000000003149 PMC11090028

[6] de C Williams AC Nicholas MK Richardson PH Pither CE Fernandes J. Generalizing from a controlled trial: the effects of patient preference versus randomization on the outcome of inpatient versus outpatient chronic pain management. PAIN 1999;83:57–65. 10506672 10.1016/s0304-3959(99)00074-3

[7] Dear BF Gandy M Karin E Staples LG Johnston L Fogliati VJ Wootton BM Terides MD Kayrouz R Perry KN Sharpe L Nicholas MK Titov N. The pain course: a randomised controlled trial examining an internet-delivered pain management program when provided with different levels of clinician support. PAIN 2015;156:1920–35. 26039902 10.1097/j.pain.0000000000000251 PMC4770347

[8] Dear BF Karin E Fogliati R Dudeney J Nielssen O Scott AJ Gandy M Bisby MA Heriseanu AI Hathway T Staples L Titov N Schroeder L. A cost-effectiveness analysis of an internet-delivered pain management program delivered with different levels of clinician support: results from a randomised controlled trial. J Pain 2021;22:344–58. 33227510 10.1016/j.jpain.2020.11.003

[9] Department of Health and Aged Care. In: AGDo Health editor, ed. Medicare benefits schedule book. Oxford: Department of Health and Aged Care; 2019:Vol. 2020.

[10] Drummond MS Torrance GW O’Brien BJ Stoddart GL. Methods for the economic evaluation of health care programme. 3rd ed. Oxford: Oxford University Press; 2005.

[11] Elbers S Wittink H Konings S Kaiser U Kleijnen J Pool J Köke A Smeets R. Longitudinal outcome evaluations of interdisciplinary multimodal pain treatment programmes for patients with chronic primary musculoskeletal pain: a systematic review and meta-analysis. Eur J Pain 2022;26:310–35. 34624159 10.1002/ejp.1875 PMC9297911

[12] Fordyce WE Fowler RS Jr Lehmann JF Delateur BJ Sand PL Trieschmann RB. Operant conditioning in the treatment of chronic pain. Arch Phys Med Rehabil 1973;54:399–408. 4729785

[13] George B Harris A Mitchell A. Cost-effectiveness analysis and the consistency of decision making: evidence from pharmaceutical reimbursement in Australia (1991 to 1996). Pharmacoeconomics 2001;19:1103–9. 11735677 10.2165/00019053-200119110-00004

[14] Goossens ME Rutten-Van Mölken MP Kole-Snijders AM Vlaeyen JW Van Breukelen G Leidl R. Health economic assessment of behavioural rehabilitation in chronic low back pain: a randomised clinical trial. Health Econ 1998;7:39–51. 9541083 10.1002/(sici)1099-1050(199802)7:1<39::aid-hec323>3.0.co;2-s

[15] Hawthorne G Osborne R. Population norms and meaningful differences for the assessment of quality of life (AQoL) measure. Aust N Z J Public Health 2005;29:136–42. 15915617 10.1111/j.1467-842x.2005.tb00063.x

[16] Hedman-Lagerlöf M Hedman-Lagerlöf E Ljótsson B Wicksell RK Flink I Andersson E. Cost-effectiveness and cost-utility of internet-delivered exposure therapy for fibromyalgia: results from a randomized, controlled trial. J Pain 2019;20:47–59. 30107241 10.1016/j.jpain.2018.07.012

[17] Holm LW Carroll LJ Cassidy JD Hogg-Johnson S Côté P Guzman J Peloso P Nordin M Hurwitz E van der Velde G Carragee E Haldeman S, Bone and Joint Decade 2000-2010 Task Force on Neck Pain and Its Associated Disorders. The burden and determinants of neck pain in whiplash-associated disorders after traffic collisions: results of the bone and joint decade 2000-2010 task force on neck pain and its associated disorders. Spine 2008;33:S52–9. 18204401 10.1097/BRS.0b013e3181643ece

[18] Huffman KL Rush TE Fan Y Sweis GW Vij B Covington EC Scheman J Mathews M. Sustained improvements in pain, mood, function and opioid use post interdisciplinary pain rehabilitation in patients weaned from high and low dose chronic opioid therapy. PAIN 2017;158:1380–94. 28328578 10.1097/j.pain.0000000000000907

[19] IHPA. National hospital cost data collection report, public sector, round 23, independent hospital pricing authority (financial year 2018–19). Vol. 2021. Independent Hospital Pricing Authority.

[20] Kjellsson G Clarke P Gerdtham UG. Forgetting to remember or remembering to forget: a study of the recall period length in health care survey questions. J Health Econ 2014;35:34–46. 24595066 10.1016/j.jhealeco.2014.01.007

[21] Lamb SE Lall R Hansen Z Castelnuovo E Withers EJ Nichols V Griffiths F Potter R Szczepura A Underwood M, BeST trial group. A multicentred randomised controlled trial of a primary care-based cognitive behavioural programme for low back pain. The back skills training (BeST) trial. Health Technol Assess 2010;14:1–253. iii–iv. 10.3310/hta14410 20807469

[22] Lambeek LC Bosmans JE Van Royen BJ Van Tulder MW Van Mechelen W Anema JR. Effect of integrated care for sick listed patients with chronic low back pain: economic evaluation alongside a randomised controlled trial. BMJ 2010;341:c6414. 21118874 10.1136/bmj.c6414 PMC2995018

[23] Lamper C Huijnen IPJ Goossens M Winkens B Ruwaard D Verbunt J Kroese ME. The (cost-)effectiveness and cost-utility of a novel integrative care initiative for patients with chronic musculoskeletal pain: the pragmatic trial protocol of network pain rehabilitation Limburg. Health Qual Life Outcomes 2020;18:320. 33004059 10.1186/s12955-020-01569-9 PMC7528600

[24] Landén Ludvigsson M Peolsson A Peterson G Dedering Å Johansson G Bernfort L. Cost-effectiveness of neck-specific exercise with or without a behavioral approach versus physical activity prescription in the treatment of chronic whiplash-associated disorders: analyses of a randomized clinical trial. Medicine 2017;96:e7274. 28640136 10.1097/MD.0000000000007274 PMC5484244

[25] Lee YY Mihalopoulos C Chatterton ML Fletcher SL Chondros P Densley K Murray E Dowrick C Coe A Hegarty KL Davidson SK Wachtler C Palmer VJ Gunn JM. Economic evaluation of the Target-D platform to match depression management to severity prognosis in primary care: a within-trial cost-utility analysis. PLoS One 2022;17:e0268948. 35613149 10.1371/journal.pone.0268948 PMC9132336

[26] McCabe C Claxton K Culyer AJ. The NICE cost-effectiveness threshold: what it is and what that means. Pharmacoeconomics 2008;26:733–44. 18767894 10.2165/00019053-200826090-00004

[27] Neumann PJ Goldie SJ Weinstein MC. Preference-based measures in economic evaluation in health care. Annu Rev Public Health 2000;21:587–611. 10884966 10.1146/annurev.publhealth.21.1.587

[28] Neumann PJ Cohen JT Weinstein MC. Updating cost-effectiveness--the curious resilience of the $50,000-per-QALY threshold. N Engl J Med 2014;371:796–7. 25162885 10.1056/NEJMp1405158

[29] New South Wales-State Insurance Regulatory Authority. Fees and rates. Australia: SIRA: NSW Government and NSW; 2019:2020.

[30] NICE. Chronic pain (primary and secondary) in over 16s: assessment of all chronic pain and management of chronic primary pain; NICE guideline [NG193]. Vol. 2023. UK: The National Institute of Health and Care Excellence (NICE), 2021. 33939353

[31] Nicholas MK Asghari A Sharpe L Brnabic A Wood BM Overton S Tonkin L de Sousa M Finniss D Beeston L Sutherland A Corbett M Brooker C. Cognitive exposure versus avoidance in patients with chronic pain: adherence matters. Eur J Pain 2014;18:424–37. 23939595 10.1002/j.1532-2149.2013.00383.x

[32] Nicholas MK Costa DSJ Blanchard M Tardif H Asghari A Blyth FM. Normative data for common pain measures in chronic pain clinic populations: closing a gap for Clinicians and researchers. PAIN 2019;160:1156–65. 30694928 10.1097/j.pain.0000000000001496

[33] Nicholas MK Asghari A Sharpe L Beeston L Brooker C Glare P Martin R Molloy A Wrigley PJ. Reducing the use of opioids by patients with chronic pain: an effectiveness study with long-term follow-up. PAIN 2020;161:509–19. 31764391 10.1097/j.pain.0000000000001763

[34] Paganini S Lin J Kählke F Buntrock C Leiding D Ebert DD Baumeister H. A guided and unguided internet- and mobile-based intervention for chronic pain: health economic evaluation alongside a randomised controlled trial. BMJ Open 2019;9:e023390. 10.1136/bmjopen-2018-023390 PMC6500312 30967405

[35] Raftery JP. Paying for costly pharmaceuticals: regulation of new drugs in Australia, England and New Zealand. Med J Aust 2008;188:26–8. 18205559 10.5694/j.1326-5377.2008.tb01500.x

[36] Richardson J Iezzi A Khan MA Maxwell A. Validity and reliability of the assessment of quality of life (AQoL)-8D multi-attribute utility instrument. Patient 2014;7:85–96. 24271592 10.1007/s40271-013-0036-x PMC3929769

[37] Richardson J Sinha K Iezzi A Khan MA. Modelling utility weights for the assessment of quality of life (AQoL)-8D. Qual Life Res 2014;23:2395–404. 24719017 10.1007/s11136-014-0686-8

[38] Richardson J Iezzi A Khan MA. Why do multi-attribute utility instruments produce different utilities: the relative importance of the descriptive systems, scale and 'micro-utility' effects. Qual Life Res 2015;24:2045–53. 25636660 10.1007/s11136-015-0926-6 PMC4493939

[39] Townsend CO Kerkvliet JL Bruce BK Rome JD Hooten MW Luedtke CA Hodgson JE. A longitudinal study of the efficacy of a comprehensive pain rehabilitation program with opioid withdrawal: comparison of treatment outcomes based on opioid use status at admission. PAIN 2008;140:177–89. 18804915 10.1016/j.pain.2008.08.005

[40] Wang H Zhao H. A study on confidence intervals for incremental cost-effectiveness ratios. Biom J 2008;50:505–14. 18663759 10.1002/bimj.200810439

[41] Wang S Gum D Merlin T. Comparing the ICERs in medicine reimbursement submissions to NICE and PBAC-does the presence of an explicit threshold affect the ICER proposed? Value Health 2018;21:938–43. 30098671 10.1016/j.jval.2018.01.017

[42] Wayne PMB JE Eisenberg DM Osypiuk K Gow BJ Davis RB Witt CM Reinhold T. Cost-effectiveness of a team-based integrative medicine approach to the treatment of back pain. J Altern Complement Med 2019;25:S138–46. 30870015 10.1089/acm.2018.0503 PMC6444892

[43] Williams A van Dongen JM Kamper SJ O'Brien KM Wolfenden L Yoong SL Hodder RK Lee H Robson EK Haskins R Rissel C Wiggers J Williams CM. Economic evaluation of a healthy lifestyle intervention for chronic low back pain: a randomized controlled trial. Eur J Pain (London, England) 2019;23:621–34. 10.1002/ejp.1334 30379386

[44] Wright DR Haaland WL Ludman E McCauley E Lindenbaum J Richardson LP. The costs and cost-effectiveness of collaborative care for adolescents with depression in primary care settings: a randomized clinical trial. JAMA Pediatr 2016;170:1048–54. 27654449 10.1001/jamapediatrics.2016.1721

